# A Direct Aspiration First Pass Technique for Mechanical Thrombectomy in the Setting of a Suspected Cerebral Aneurysm

**DOI:** 10.7759/cureus.2254

**Published:** 2018-03-01

**Authors:** Ali S Haider, Suraj Sulhan, Dean Leonard, Haris Rana, Umair Khan, Tijani Osumah, Steven Vayalumkal, Richa Thakur, Kennith F Layton

**Affiliations:** 1 Texas A&M College of Medicine; 2 School of Medicine, Ross University; 3 School of Medicine, St. George's University; 4 Department of Urology, Mayo Clinic; 5 Department of Radiology, Baylor University Medical Center

**Keywords:** ischemic stroke, aneurysm, endovascular neurosurgery, interventional neuroradiology, interventional neurology

## Abstract

There is little guidance in the literature on which thrombectomy technique is preferred in patients with acute ischemic stroke and concomitant aneurysms. Here, we present the case of a 58-year-old female with an acute ischemic stroke requiring emergent thrombectomy that was complicated by the presence of multiple, nonruptured intracranial aneurysms. Imaging confirmed an occlusion of the right middle cerebral artery and multiple nonruptured intracranial aneurysms. The patient was administered intravenous recombinant tissue plasminogen activator and the thrombus was aspirated via a direct aspiration first pass technique (ADAPT). Her symptoms improved significantly postoperatively with a consequent National Institutes of Health Stroke Scale (NIHSS) score of 0. The purpose of this case report is to give an overview and compare various techniques that can help guide the physician for safe, early revascularization while reducing recanalization time in patients having an ischemic stroke who also harbor intracranial aneurysms.

## Introduction

Combined ischemic and hemorrhagic strokes are the fifth leading cause of death in the United States, affecting approximately 795,000 people each year and costing about $40 billion annually [1]. It accounts for 50% of stroke patients’ long-term disability, requiring assistance and discharge to rehabilitation or skilled nursing facilities [2]. Until recently, intravenous recombinant tissue-type plasminogen activator (IV-rtPA) was the only established therapeutic option [3]. Endovascular approaches using stent retrievers and aspiration devices have since gained momentum in establishing revascularization and are now considered standard-of-care for selected patients with emergent large vessel occlusions (ELVO). However, a novel technique using the newest generation of large-bore aspiration catheters, known as a direct aspiration first pass technique (ADAPT), is a direct aspiration first-pass technique that has also been reported [4]. In comparing the cost, duration, and efficacy of the stent retriever and ADAPT techniques, it is still unclear which treatment shows better clinical outcomes, warranting the need for additional trials to establish a guideline. There are few clinical trials underway that will provide further insight into the differences between direct aspiration and stent retriever thrombectomy [5]. Here, we present a unique case of an M1 segment right middle cerebral artery (MCA) occlusion harboring a hidden, bilobed, unruptured aneurysm and a choice of whether to use a stent retriever or the ADAPT technique in establishing revascularization.

## Case presentation

**Figure 1:**
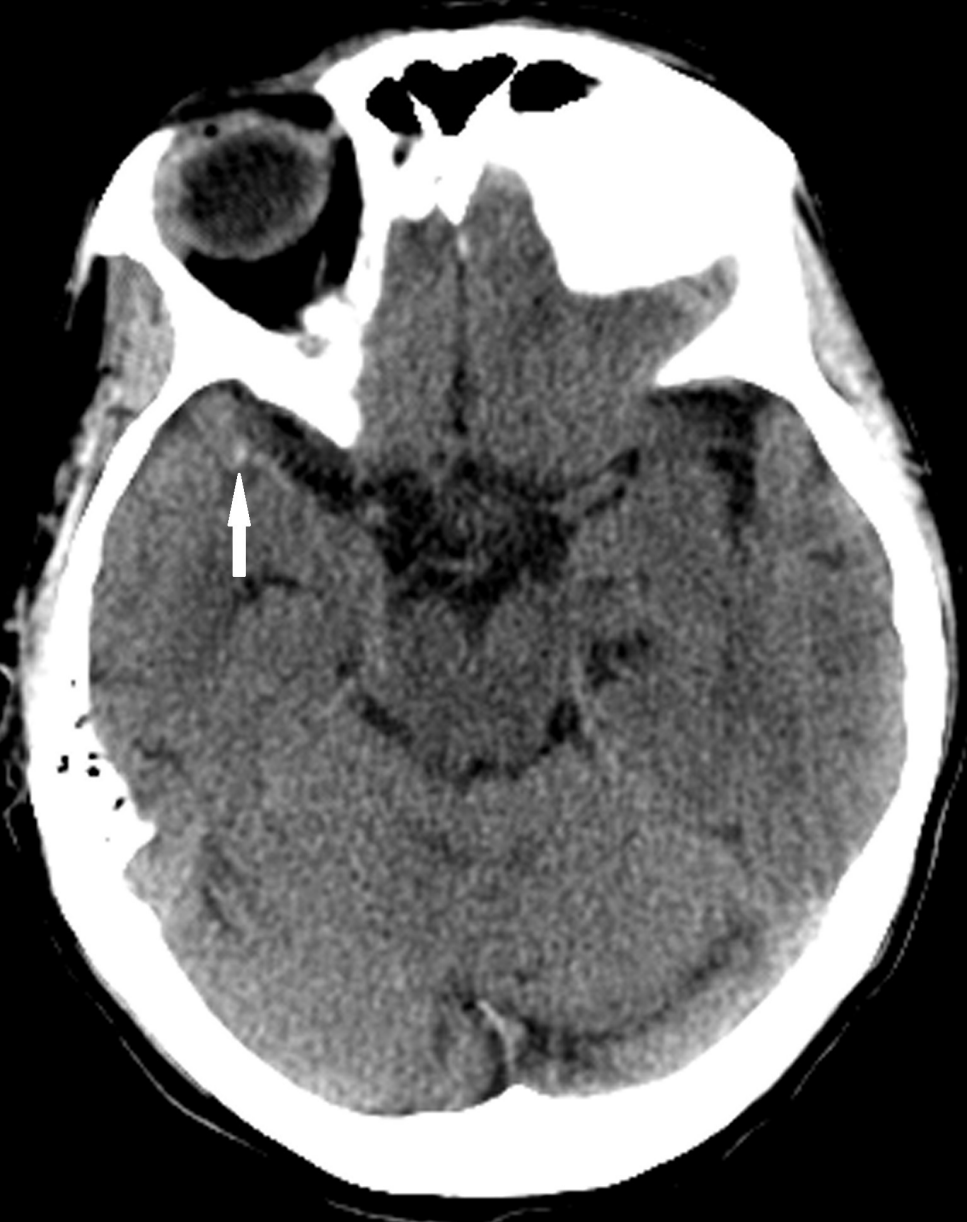
Noncontrast head computed tomography demonstrates markedly asymmetric density in the distal right middle cerebral artery (arrow) compatible with the dense middle cerebral artery sign

A 58-year-old woman with a history of chronic obstructive pulmonary disease, hypertension, and smoking presented to the emergency department two hours after the onset of severe left-sided weakness with facial droop, dysarthria, right gaze preference, and weakness. Her initial National Institutes of Health Stroke Scale (NIHSS) was 8 by the tele-neurology exam using the spoke and hub robot. The patient was administered intravenous rtPA (IV-rtPA) and transferred to our high-volume comprehensive stroke center for endovascular stroke therapy. The initial head computerized tomography (CT) demonstrated a dense MCA sign on the right (Figure 1).

**Figure 2:**
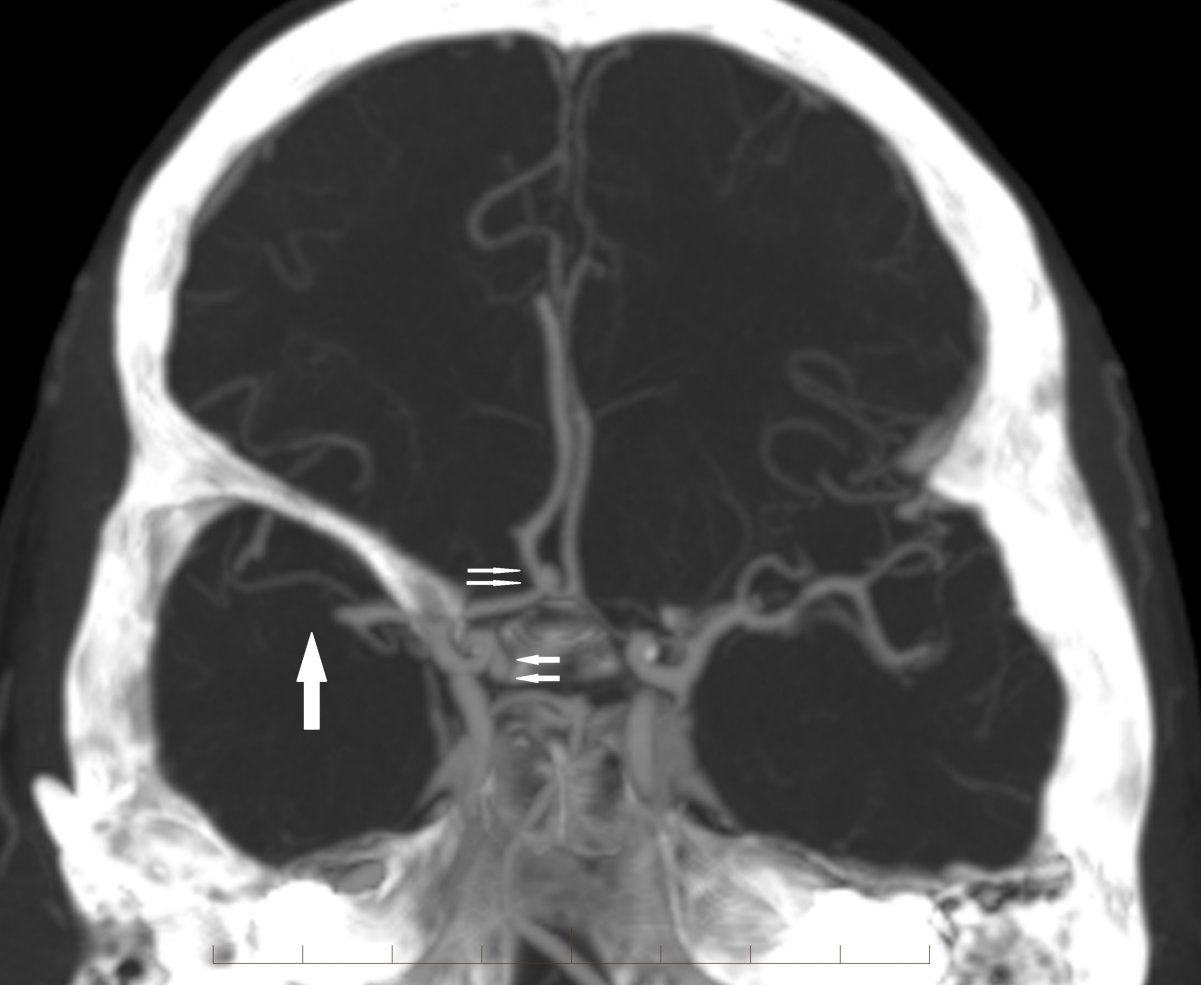
Coronal reconstructed image from the computed tomography angiogram confirms the right M1 segment middle cerebral artery occlusion (arrow). Also noted are the right superior hypophyseal and anterior communicating artery aneurysms (double arrows)

CT angiography confirmed an occlusion of the M1 segment of the right middle cerebral artery in addition to nonruptured anterior communicating (ACOM) and right superior hypophyseal aneurysms (Figure 2).

**Figure 3:**
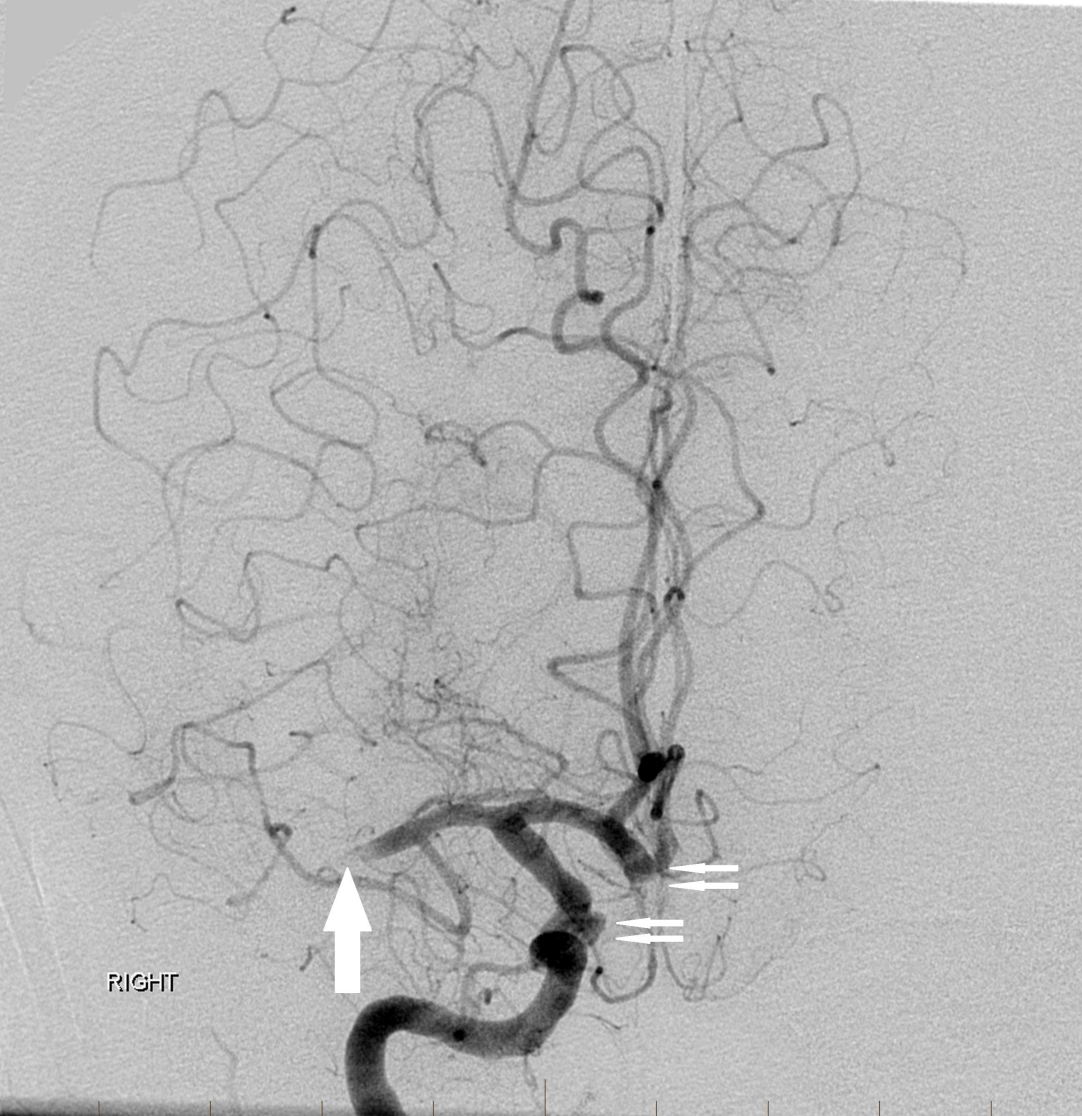
Initial digital subtraction angiography image from a right internal carotid artery angiogram again reveals an M1 middle cerebral artery occlusion (arrow) on the right with leptomeningeal collaterals. Also confirmed are the right superior hypophyseal and anterior communicating artery aneurysms (double arrows) "Right" indicates the patient's right side.

An initial catheter angiography confirmed the occlusion of the M1 segment right MCA with good leptomeningeal collaterals (Figure 3).

**Figure 4:**
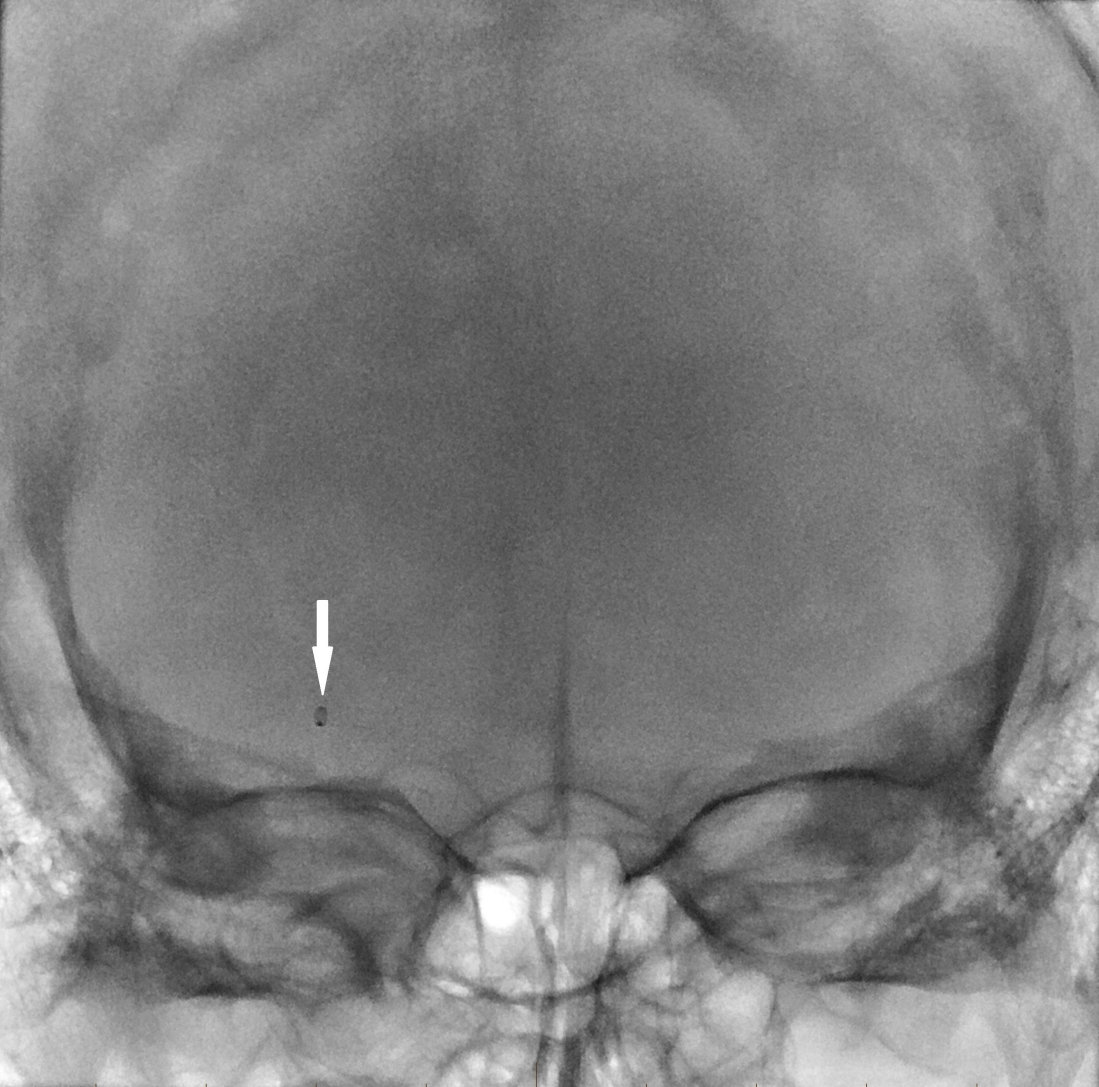
Digital angiogram native image showing the ACE 68 aspiration catheter (Penumbra Inc., CA, US) with the tip (arrow) proximal to the clot in the M1 segment of the right middle cerebral artery

Given the presence of the clot at the MCA bifurcation and aneurysms of the right superior hypophyseal and anterior communicating locations, a decision was made to stay proximal and use ADAPT (Figure 4).

**Figure 5:**
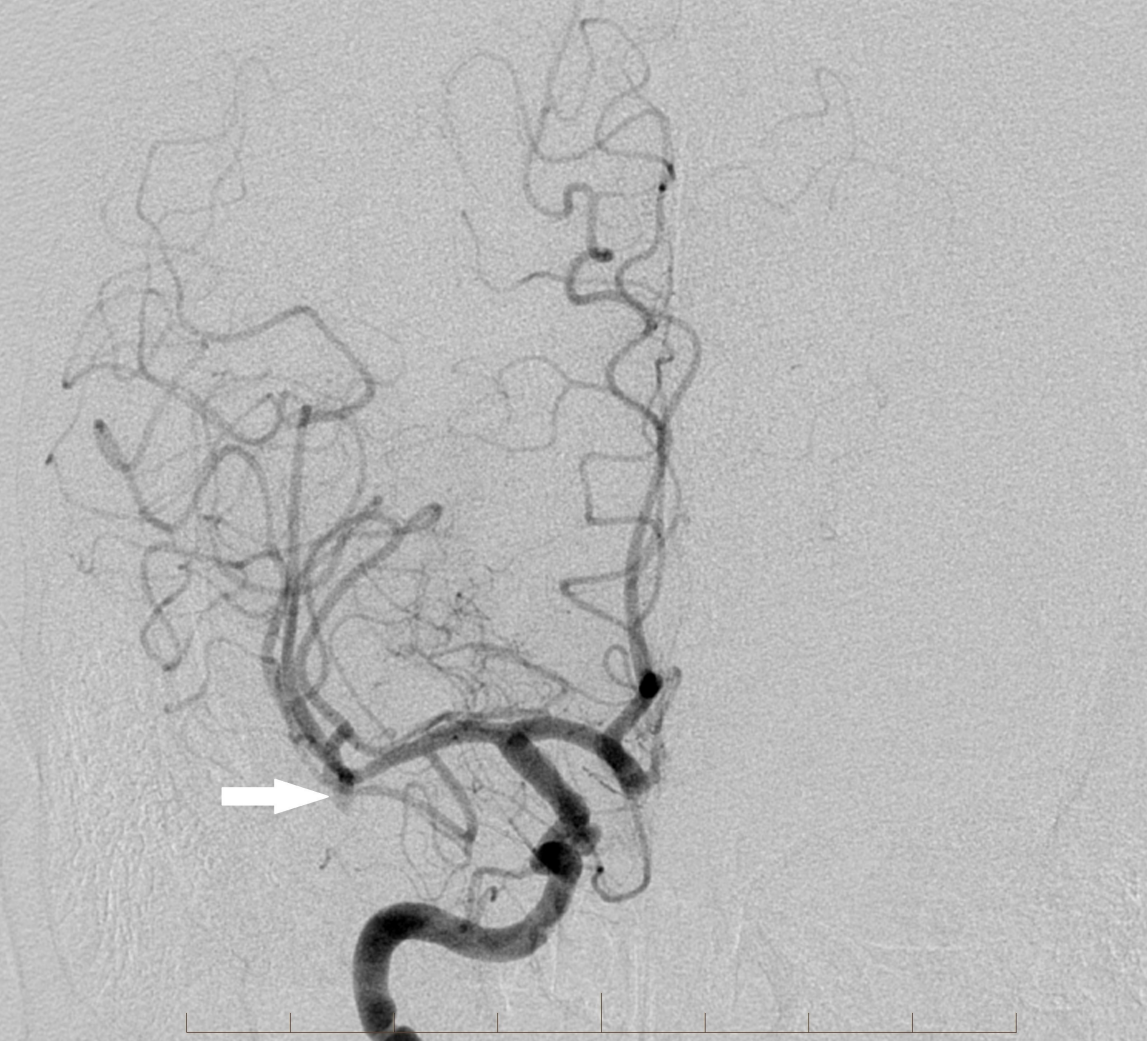
Post-thrombectomy right internal carotid artery angiography documents excellent recanalization of the right middle cerebral artery. There is a bilobed aneurysm present at the right middle cerebral artery bifurcation (arrow). Traversing this aneurysm was avoided by the use of a direct aspiration first pass technique (ADAPT)

This technique was chosen because it avoids the need for manipulating a stent retriever blindly through the MCA bifurcation in a patient with a higher risk of harboring an additional unidentified aneurysm at the MCA bifurcation. After successfully aspirating the large clot fragment using ADAPT, a bilobed irregular aneurysm was found at the right MCA bifurcation (Figure 5).

Recanalization with ADAPT showed an improvement from 0 to 2b on the modified thrombolysis in cerebral infarction (TICI) scale. The patient showed dramatic improvement on the angiography table with a postoperative NIHSS score of 0. The patient was clinically suitable for discharge and sent home after five days with the plan to follow up with the department of neurosurgery to discuss possible future treatments of her multiple nonruptured cerebral aneurysms.

## Discussion

A recent meta-analysis of acute ischemic stroke treatment showed that patients randomized to stent retriever therapy with IV-rtPA had significantly improved rates of functional independence at 90 days compared with those randomized to IV-rtPA alone [6]. Additionally, the introduction of ADAPT dramatically improved the efficacy of aspiration thrombectomy, as it utilizes a flexible and atraumatic large-bore aspiration catheter that can access the intracranial vasculature and provide a large cross-sectional area for thrombus aspiration without having to completely traverse the occlusion [4]. One study demonstrated that ADAPT was able to establish revascularization in 78% of cases alone and up to 95% of cases with adjunctive devices [4]. However, compared with stent retriever data, ADAPT showed similar rates of good functional outcome and mortality, thus making the decision on which to use less clear [4]. Another recent study showed dramatically improved clinical outcomes and recanalization rates for the Sol-Arc technique, which used a Solitaire stent retriever in conjunction with the Arc catheter (Medtronic, Minneapolis, MN) for locally assisted aspiration stent retriever thrombectomy. The authors reported a 92% successful recanalization rate and no complications; 42% of these patients were discharged home with an NIHSS of 0 and 67% of the patients had a favorable modified Rankin Scale (mRS) of 0-2 at 90 days [7]. Our case was unique in that the patient had multiple known and suspected unknown aneurysms, prompting a less traumatic approach to remove the clot at the right MCA bifurcation. While there are cases in the literature describing stent retriever thrombectomy in the setting of an intracranial aneurysm without complications, there are also case reports of rupture related to the use of stent retrievers [8-10]. While aspiration thrombectomy may be safer than stent retriever thrombectomy in the setting of an unruptured intracranial aneurysm, there are no studies available comparing these two techniques for ischemic stroke therapy. Although aspiration thrombectomy sometimes requires passing a microwire and microcatheter through the occluded segment for support, it can often be done while remaining entirely proximal to the occluded segment. Our presumption that aspiration thrombectomy is safer in this clinical scenario is theoretical and deserves further investigation in larger studies.

## Conclusions

Stent retrievers and aspiration devices are two successful endovascular approaches for mechanical thrombectomy in the setting of acute ischemic stroke. The decision to use one device over the other is sometimes difficult and dependent on factors such as cost, ease of use, and invasiveness. Our case highlights one of many other possible factors that require additional studies to establish clear guidelines that a neurointerventional surgeon may use in establishing correct treatment for the patient.
